# Papillion-Lefèvre Syndrome: Periodontists' Perspective

**DOI:** 10.1155/2015/137381

**Published:** 2015-10-08

**Authors:** Sunil Kumar Biraggari, K. Krishna Mohana Reddy, J. Sudhakar, Shiva Shankar Bugude, Rajesh Nichenametla, Mazher Ahmed Hakeem, Swati Reddy Tiyyagura

**Affiliations:** G Pulla Reddy Dental College & Hospital, India

## Abstract

Papillion-Lefèvre Syndrome is a very rare disorder of autosomal recessive inheritance distinguished by palmar plantar hyperkeratosis and early onset of periodontitis affecting the dentition. Genetic studies have identified a mutation in the major gene locus of chromosome 11q14 with loss of function. Cathepsin C gene is to be responsible for Papillion-Lefèvre Syndrome. The present case report describes a 13-year-old female, who visited the Department of Periodontology with the chief compliant of bleeding gums and loose teeth. She presented with the signs and symptoms of Papillion-Lefèvre Syndrome. The patient had premature shedding of her deciduous dentition. On clinical examination, extraorally, the patient presented with persistent thickening, flaking, and scaling of the skin of palms and soles. Her intraoral examination revealed gingival inflammation, abscess formation, and periodontal pockets. Her intraoral radiographs showed bone loss involving the central incisors and molars. The patient underwent periodontal therapy and is under maintenance.

## 1. Introduction

The Papillion-Lefèvre Syndrome (PLS) was first described by Papillon and Lefèvre in the year 1924 [1]. PLS is a rare autosomal recessive disorder distinguished by severe aggressive periodontitis of both deciduous and permanent dentition and associated with palmoplantar hyperkeratosis [2]. It has a very low prevalence rate of 1 in 4 million people. There are 2 to 4 heterozygotes for the disease out of 1000 people [3]. There is equal gender distribution and no racial predominance present [4]. Parental consanguinity is demonstrated in 20% to 40% of patients [3]. Symptoms of PLS can be evident as early as 2 months of age with the appearance of hyperkeratotic lesions of the hands and feet. Both the deciduous and permanent dentitions are affected, resulting in premature tooth loss [4]. Other findings may include psoriasis like plaques of the elbows and knees, dystrophic nails, intracranial calcification, and recurrent cutaneous infection. Besides these features, PLS patients can manifest with increased incidence of pyogenic infections, liver abscesses, or mental retardation as its complications [5].

The etiological basis for PLS was long debated owing to its rare occurrence. The occurrence of PLS has been implicated to mutations of Cathepsin C gene (CTSC gene). The PLS locus has been mapped to the chromosome 11q14–q21 [6]. The CTSC gene consists of seven exons and encodes a lysosomal cysteine protease or dipeptidyl aminopeptidase I (DPPI), which functions by removing dipeptides from the amino terminus of the protein substrate. It is present in most tissues of the body and is highly expressed in cells of the immune system (polymorphic nuclear leukocytes, alveolar macrophages, and their precursors). These cells are associated with a wide range of immune and inflammatory processes, including cell-mediated cytotoxicity, phagocytic destruction of bacteria, and activation and deactivation of cytokines. Additionally, DPPI is thought to play an important role in protein degradation and proenzyme activation. Therefore, it is likely that any effective change in the coding sequences of the Cathepsin C gene or its allele may result in a defective or truncated protein with improper function, which ultimately results in abolished activity in the homozygous patients compared to those with heterozygous genetic makeup. Moreover, the Cathepsin C gene is highly expressed in the epithelial regions such as keratinized oral gingiva, palms, soles, and knees. These are generally the areas that are most commonly affected by PLS [7]. The pathogenic mechanisms behind the manifestation of the various symptoms of PLS remain elusive. An impaired chemotactic and phagocytic function of polymorphonuclear leukocytes (PMNs) and impaired reactivity to T and B cell mitogens had also been reported [8].

Similar to the dermatologic manifestations, periodontal manifestations also begin during early childhood. The primary teeth erupt at the expected age, in the normal eruption pattern, and are of normal anatomic form and structure. Eruption of the primary dentition is typically accompanied by severe gingivitis and generalized aggressive periodontitis. The primary teeth frequently become loose and exfoliate by the age of 5. With loss of the dentition, gingival inflammation resides. With the eruption of the permanent teeth, the same cycle of events unfold and, without intervention, the permanent teeth may be lost within a few years [9].

Treatment protocols consisting of conventional therapy with oral hygiene instructions, professional scaling and root planing, frequent recalls, and antibiotics have been reported successful in preventing tooth loss in PLS patients [10]. Identification of specific periodontal pathogens, appropriate antibiotic coverage, and use of oral retinoids may improve the viability of the existing teeth although better treatment modalities should also be developed.

We hereby report a 13-year-old girl with PLS. The present case report is aimed at describing the features of PLS and a detailed review of literature.

## 2. Case History

A female patient aged 13 years was referred to the Department of Periodontics, G. Pulla Reddy Dental College and Hospital, Kurnool, with the chief complaint of loose upper front teeth along with bleeding gums since 2 years. Her past medical history revealed that she has been suffering from recurrent skin rashes and keratotic patches on her elbows, knees, and soles of feet since the age of 4 years. These symptoms worsened during the winter season. Her past dental history revealed immature exfoliation of deciduous tooth. The family history revealed consanguineous marriage of her parents. Pregnancy and delivery were normal. She has an elder sibling who also has the same keratotic lesions but of a milder expression.

### 2.1. Clinical Examination

**Figure 1:**
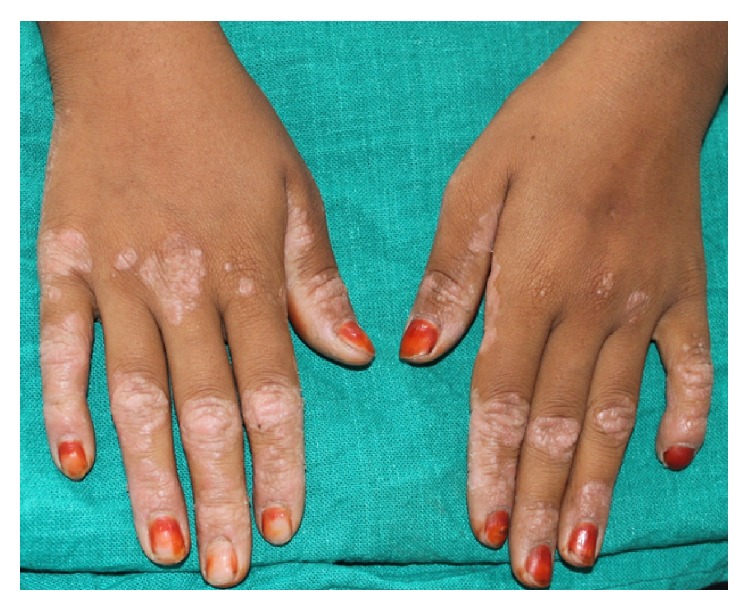
Keratotic plaques over the dorsal surface of the palms.

**Figure 2:**
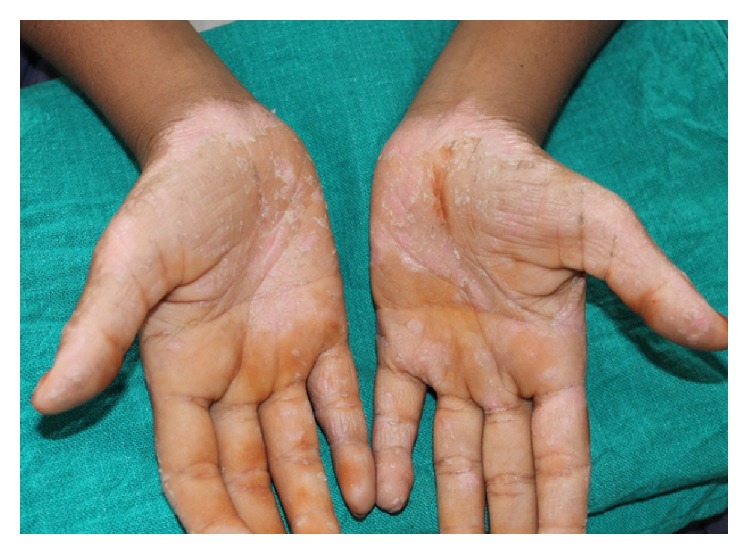
Keratotic plaques which peel off leaving underlying reddish areas over the ventral surface of the skin.

**Figure 3:**
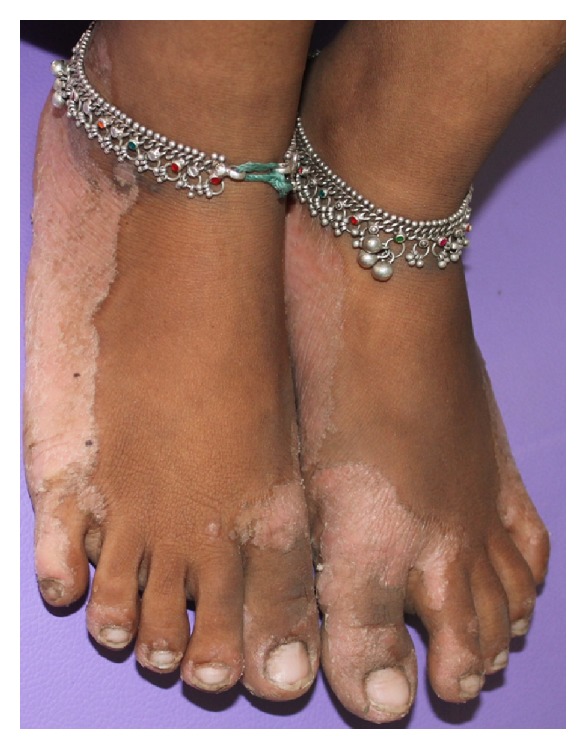
Peeling off of the skin over the feet.

Extraoral examination of the patient revealed well demarcated yellowish, keratotic lesions over the skin over palms and soles extending onto the dorsal surfaces. Skin of both palms and soles was peeling off, leaving an underlying red shiny area (Figures 1, 2, and 3). Similar lesions were also present on the knees bilaterally. Incompetent lips were evident.

**Figure 4:**
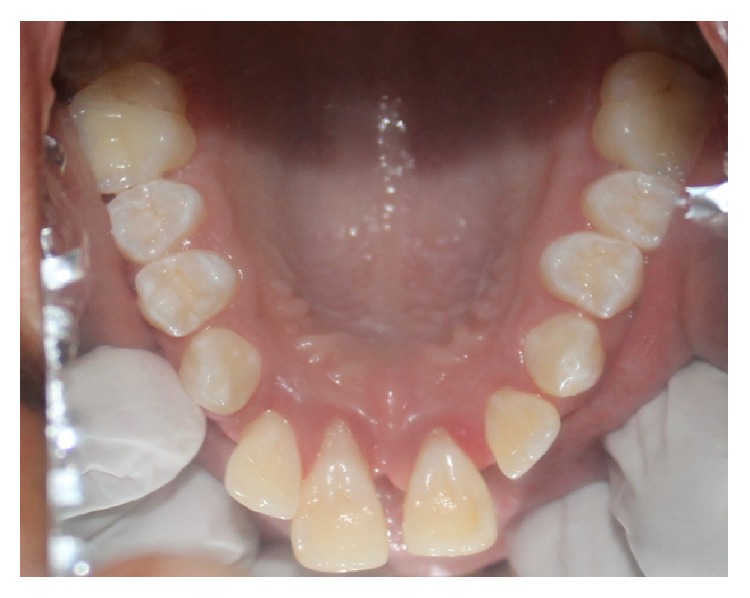
Intraoral picture showing maxillary arch with spacing of teeth between 11, 12, 13, 21, 22, and 23.

**Figure 5:**
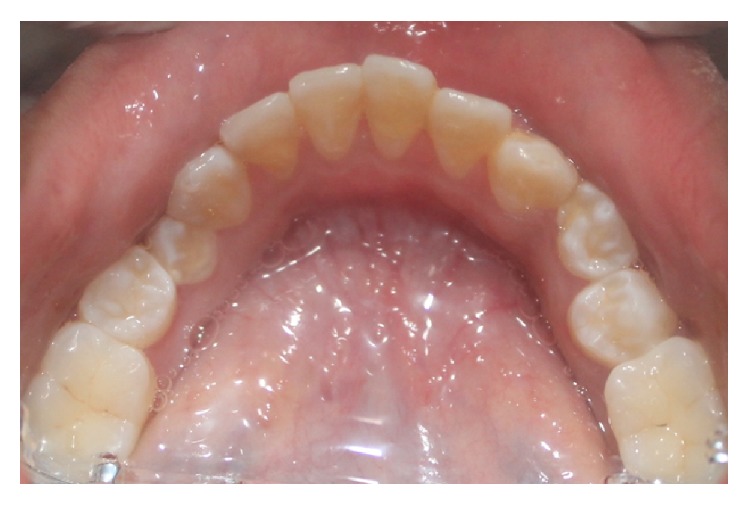
Intraoral picture showing mandibular arch with distoangular rotation of 34 and mesioangular rotation of 44.

**Figure 6:**
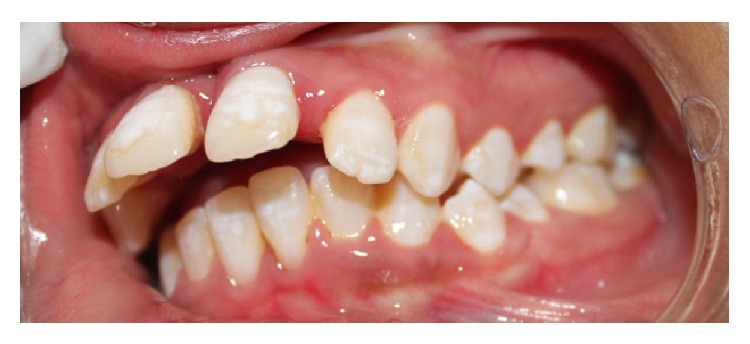
Intraoral picture showing proclination of upper anteriors.

Intraorally, the patient presented 28 teeth with proclination of 21 and 11, spacing in relation to 11, 12, 13, 21, 22, and 23, rotations in relation to 44, 35 (Figures 4 and 5), erupting teeth in relation to 44, a smooth diffuse swelling of the gingiva in relation to 36 and 37, and increased mobility and apical shift in position of gingival margin in relation to 11, 21, and 41. A detailed periodontal examination was carried out. The plaque index and gingival index were assessed. The probing depths and clinical attachment level were measured using Williams periodontal probe. For measuring the clinical attachment level, cementoenamel junction was used as a reference. There was generalized bleeding on probing and deep periodontal pockets especially in relation to 11, 21, 36, 37, 41, and 46 (Figure 6). Halitosis was present.

### 2.2. Radiographic Examination

**Figure 7:**
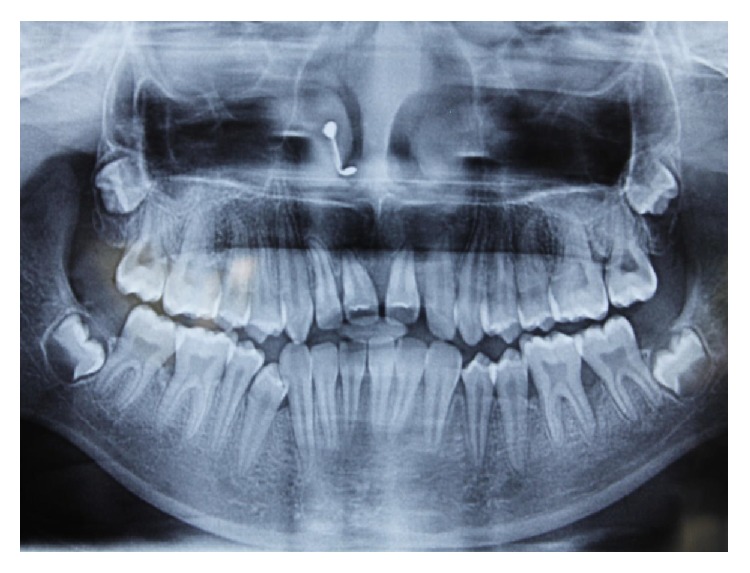
Orthopantomograph of the patient.

**Figure 8:**
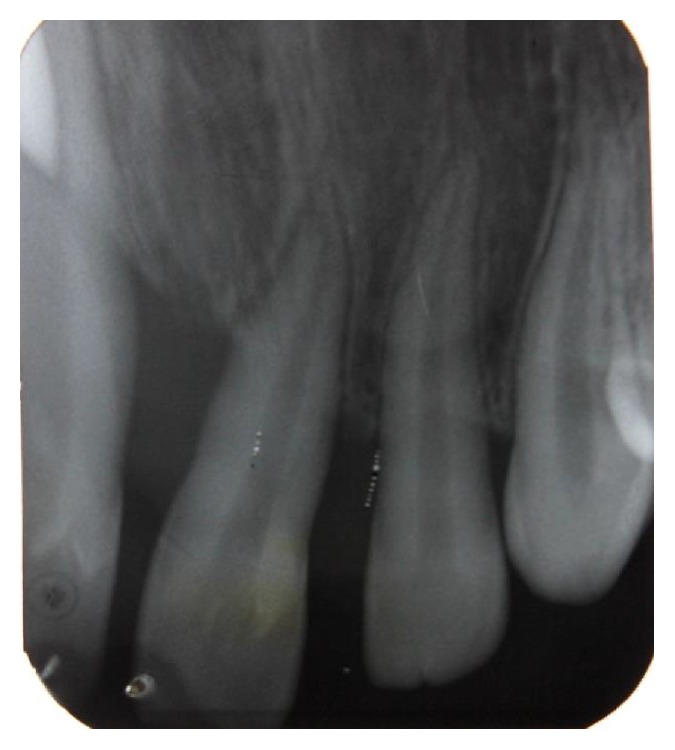
Intraoral periapical radiograph showing bone loss in relation to 11, 12.

**Figure 9:**
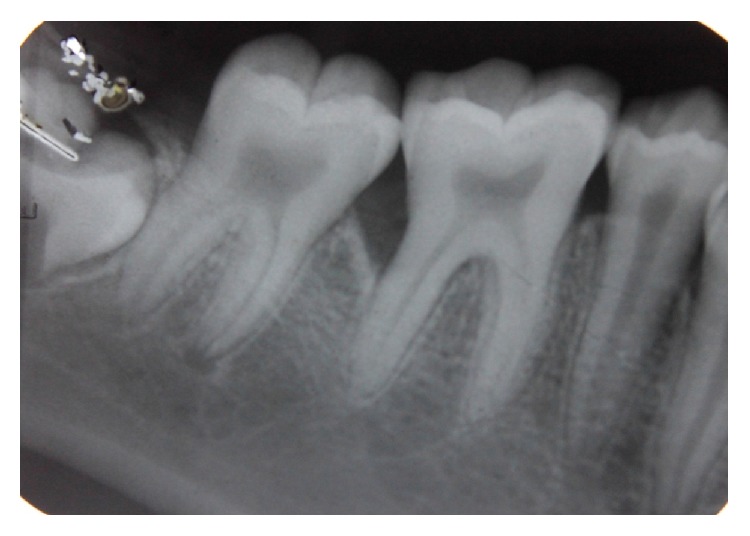
Intraoral periapical radiograph showing vertical bone loss in relation to 46.

**Figure 10:**
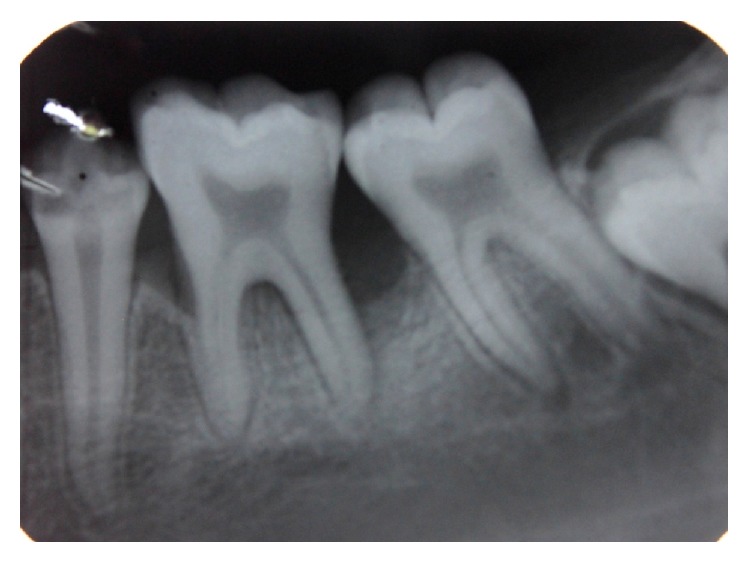
Intraoral periapical radiograph showing vertical bone loss in relation to 36.

Radiographic examination was done with the help of an orthopantomogrpah and full mouth intraoral periapical radiographs. There was vertical bone loss in relation to 11, 21, 36, 37, 41, and 46 (Figures 7, 8, 9, and 10).

### 2.3. Periodontal Therapy

Conventional periodontal therapy in the form of scaling and root planing under local anesthesia was done. Systemic antibiotics prescribed to the patient were amoxicillin 250 mg TID and metronidazole 250 mg BID which were prescribed to the patient for one week. Oral hygiene instructions were given to the patient and she was also advised to use Chlorhexidine mouth rinse. As prescribed by the dermatologist, she was advised to use Acutret 10 mg daily once for her skin lesions.

**Figure 11:**
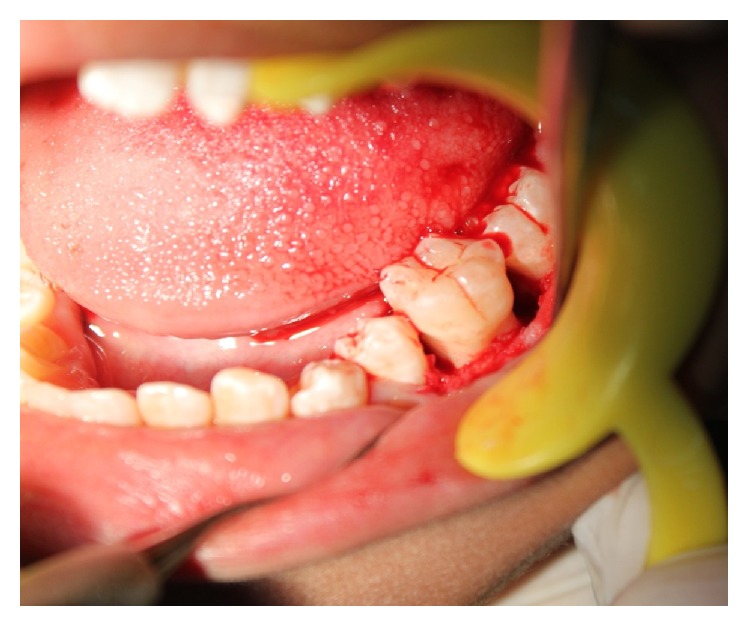
Surgical exploration in relation to 36.

**Figure 12:**
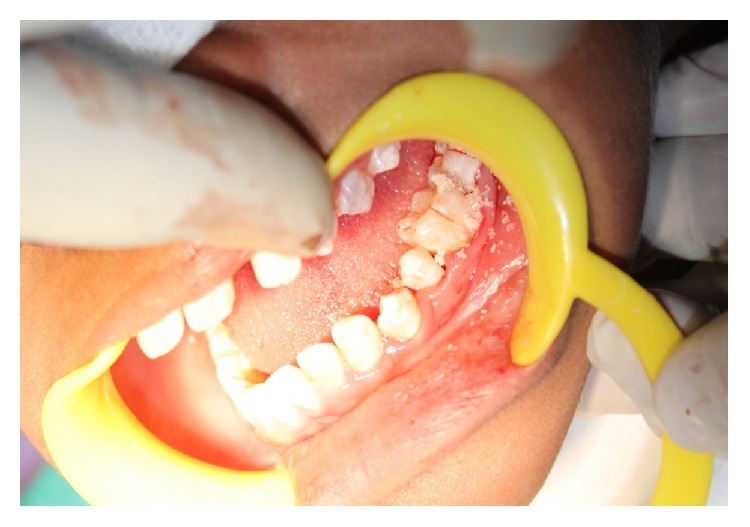
Placement of osseograft in relation to 36.

The patient was recalled after one week and one month and during every visit her periodontal status was evaluated. Based on her clinical and radiographic features, further treatment comprised of surgical periodontal therapy with regenerative periodontal procedures using osseograft in relation to the vertical defect of 36 and 46 (Figures 11 and 12). Patient was under proper antibiotic coverage. Patient was recalled after one month, 3 months, 6 months, and 12 months after surgical periodontal therapy. There was gradual reduction in probing depths and improvement in bleeding on probing and general oral hygiene of the patient. The patient is under regular followup.

## 3. Discussion

Papillion-Lefèvre Syndrome is a very rare genodermatosis of autosomal recessive inheritance. It is a condition characterized by diffuse transgradient palmoplantar hyperkeratosis and rapidly progressive and destructive periodontitis, affecting both primary and permanent dentition. It usually commences between 2 months of age to 4 years. Clinically, patients present with bleeding gums, gingivitis, erythema, and suppuration which eventually leads to tooth loss. PLS can thus affect the morale of the growing children and have an adverse effect on them [4]. Therefore, an early dental intervention and parental counseling are essential to provide a multidisciplinary treatment approach for the prognosis and betterment of the quality of life of these children [11].

The exact pathogenesis behind PLS remains obscure, though a combination of genetic, immunologic, and microbiological bases has been proposed [12]. The evolving sophistication in genomic science has implicated the protein Cathepsin C as the key player underlying the pathogenesis of PLS. The association between Cathepsin C gene alterations and susceptibility of PLS patients to periodontitis may be explained by the role of Cathepsin C gene in influencing epithelial differentiation or desquamation [13]. As the sulcular epithelium and junctional epithelium represent the first line of defense, their aberrant differentiation may alter the mechanical barrier to periodontal pathogens. Microbiological studies have suggested a variety of subgingival microflora behind the pathogenesis of PLS [14]. The frequently identified species were* Aggregatibacter actinomycetemcomitans, Porphyromonas gingivalis,* and* Prevotella intermedia.* Among these,* Aggregatibacter actinomycetemcomitans* was detected in high frequency in the patients with PLS and was associated with the pronounced bone loss of periodontal tissue in these patients [15]. A chemotaxis is behind the destructive periodontitis in PLS patients though there was a normal neutrophil count. The decrease in the chemotaxis may be due to a cell defect in the receptor located at the surface of the neutrophils resulting in deficiencies in the cellular adhesion molecules. The most complete defect in neutrophils in such patients is a distinct molecular defect of adhesion of leukocytes related to deficiency in CD11 molecule [8]. Therefore, a combined genetic, immunologic, and microbial pathway may be involved wherein mutation of Cathepsin C gene is responsible for the microbial induced inflammatory response [12]. As a result, it is likely that inflammatory infiltrate at local sites of periodontal infection is not under normal regulatory control, and accentuated influx of neutrophils and retention of inflammatory infiltrate and their proteases may play a significant role in continued periodontal destruction. It may be for this reason that it is difficult to control and limit periodontitis once lesions are established in PLS.

Our patient clinically showed generalized bleeding on probing, increased probing depths, and mobility of teeth along with keratotic plaques on the hands and soles of feet. Radiographical examination revealed increased bone loss. Based on her clinical and radiographic examination, her past medical and dental history, and her family history, we strongly suggest the diagnosis of PLS.

The differential diagnosis includes Haim-Munk Syndrome and Hypophosphatasia. Haim-Munk Syndrome shares similar features with Papillion-Lefèvre Syndrome, but the patients also present with arachnodactyly of the hands which is a typical feature of Haim-Munk Syndrome. Hypophosphatasia also shows palmoplantar keratodermatitis along with progressive periodontitis. But hypophosphatasia also appears with deficiency of alkaline phosphatase, the values of which are within normal limits for our patient. Other palmoplantar keratodermal diseases like Gamborg-Nielsen Syndrome, Unna-Thost Syndrome, Mal de Mala, and Howel Evans Syndrome could also be considered.

A definitive treatment protocol has still not been postulated to treat patients with PLS, though many treatment modalities have suggested treating PLS associated periodontitis. Conventional therapy with scaling and root planing, antibiotics, and oral hygiene instructions have failed to prevent tooth loss in PLS patients [16]. Age of the patient, whether the patient is presenting with mixed dentition or deciduous dentition, systemic health of the patient, and oral hygiene maintenance affect the treatment. A multifactorial approach by appropriate antibiotic therapy against the microorganisms to prevent bacteremia, extraction of the hopeless teeth which could improve the viability of the existing teeth, periodontal surgery to treat bone loss and periodontitis, and meticulous oral hygiene can improve the condition of the patient [16]. Use of oral retinoids is also suggested. We have performed professional scaling and root planing along with osseous graft placement in relation to 36 and 46 under antibiotic coverage of amoxicillin and metronidazole. The patient was also on oral retinoids like Acitretin. Frequent recalls within 1 week, 1 month, and 3 months were done to check the oral status of the patient and weekly oral prophylaxis was done. There was improved probing depth and gingivitis also resided with an overall upgrade of her periodontal condition. Altogether, these findings suggest that, by controlling periodontal microbiota with appropriate antibiotics, conventional periodontal therapy, and surgery, we can arrest periodontitis in PLS patients.

## 4. Conclusion

As dentists, we should be familiar with the etiopathogenesis and clinical features of PLS, to be able to identify PLS patients at an early stage and prevent the progression of the disease. Children with skin lesions and periodontitis should be referred to a periodontist at the earliest, so that tooth loss is prevented and missing teeth are rehabilitated to preserve the function and esthetics of the patient. In conclusion, the management of PLS is challenging and a multifactorial approach should be undertaken. Novel approaches like specific antibiotic regimens and retinoid usage can be implemented. However, with the identification of underlying genetic defects of Cathepsin C gene and better treatments targeted at the chromosomal defect, stem cell therapy should be developed which may open new horizons for treatment of PLS patients.
